# Peptidylarginine deiminase 4 -104C/T polymorphism and risk of rheumatoid arthritis: A pooled analysis based on different populations

**DOI:** 10.1371/journal.pone.0193674

**Published:** 2018-03-08

**Authors:** Jiong Hua, Weijie Huang

**Affiliations:** Department of Orthopedics, Shanghai Pu Nan Hospital, Shanghai, China; State University of New York, UNITED STATES

## Abstract

**Background:**

Many studies have analyzed the association between peptidylarginine deiminase 4 (PADI4) -104C/T polymorphism and rheumatoid arthritis (RA). However, the results are inconsistent. This meta-analysis, based on different populations, updated and reevaluated the possible associations between PADI4 -104C/T polymorphism and the susceptibility to RA.

**Methods:**

A literature search was performed on PubMed and related Chinese databases up to April 2017. The association between PADI4 -104C/T polymorphism and RA risk was evaluated by calculating pooled odds ratios (ORs) and 95% confidence intervals (CIs).

**Results:**

A total of seventeen studies, including 5,756 RA cases and 4,987 controls, were screened out. In the overall population, PADI -104C/T polymorphism was significantly associated with an increased RA risk. In this meta-analysis stratified by ethnicity, a significant association between PADI -104C/T polymorphism and RA risk was established in China and Japan.

**Conclusions:**

Our study indicated a significantly increased association between PADI -104C/T polymorphism and RA in Chinese and Japanese populations. Because most included populations in this meta-analysis were Asian, further studies are needed to elucidate whether the PADI4 -104C/T gene confers RA in other ethnic groups.

## Introduction

Rheumatoid arthritis (RA) is the most common disease of joints in adults around the world. In the United States, approximately 2.1 million people suffered from RA, which is expected to double in the next 20 years [1]. The etiology of RA is multifactorial, including inflammatory, metabolic, and mechanical factors. Previous studies suggest that age, sex, obesity, physical activity, smoking, and genetic factors are involved in the development of RA [2,3]. Genetic factors are considered to be responsible for approximately 60% of the risk of developing RA [3]. Peptidylarginine deiminase 4 (PADI4) -104C/T is one of the most widely studied genetic variant for RA among all candidate gene polymorphisms. In 2003, Suzuki et al. inventively found an association of PADI4-104C/T polymorphism with RA diseases in a Japanese population [4]. Subsequently, a number of studies have been conducted to investigate the link between PADI4-104C/T and RA in different populations. However, this relationship is still poorly understood. Whether the features and effects of this association differ between populations from different ethnic backgrounds remains unknown. We, therefore, undertook a meta-analysis to quantitatively assess the relationship between PADI4-104C/T and RA risk in different populations.

## Materials and methods

### Identification of eligible studies

All studies that investigated the association between PADI4-104C/T polymorphism and RA published before April 2017 were considered for inclusion in the meta-analysis. PubMed, Springer Link, Ovid, Chinese Wanfang Data Knowledge Service Platform, Chinese National Knowledge Infrastructure, and Chinese Biology Medicine databases were used for the literature search. The search keywords were (PADI4 or peptidylarginine deiminase 4 or -104C/T) and rheumatoid arthritis. No restrictions were placed on language, race, ethnicity, or geographic area. All references cited in the studies were also reviewed to identify additional published work not indexed by these databases. The following inclusion criteria were employed: (1) studies describing the association between PADI4 -104C/T polymorphism and RA; (2) sufficient genetypes data to calculate the odds ratio (OR). Exclusion criteria were defined as follows: (1) studies contained incomplete data, (2) case reports, (3) editorial articles, and (4) review articles and metting abstracts.

### Data extraction

Information was carefully extracted from all eligible publications independently by two authors (Hua J and Huang WJ) according to the inclusion criteria. The titles and abstracts of all potentially relevant articles were initially screened. Full articles were then scrutinized if the title and abstract were ambiguous. Disagreements were resolved through a discussion between the two authors. The following data were extracted from each study: first author’s name, publication year, ethnicity, sample size, and available genotype information with PADI4 -104C/T polymorphism.

### Statistical analysis

The association between PADI4 -104C/T polymorphism and RA risk was evaluated by calculating pooled odds ratios (ORs) and 95% confidence intervals (CIs). The meta-analysis examined the overall association of the T allele with RA risk relative to the C allele; the differences between homozygotes TT and CC, between TT and (TC + CC), and between (TT + TC) and CC were also evaluated. The heterogeneity test and Hardy-Weinberg equilibrium (HWE) expectations in the controls were assessed by Chi-square-based Q-test. In the cases with no obvious heterogeneity between studies, OR was pooled using the fixed-effects model. Otherwise, the random-effects model was used. We compared the consistency between the fixed-effects and random-effects model to assess sensitivity analysis. Funnel plot was used to assess the potential publication bias, and the Egger’s test was applied to evaluate the funnel plot asymmetry. All statistical analyses were conducted using Stata, version 12 (StataCorp LP, College Station, TX, USA). A *P-*value less than 0.05 was considered statistically significant.

## Results

### Description of the included studies

**Fig 1:**
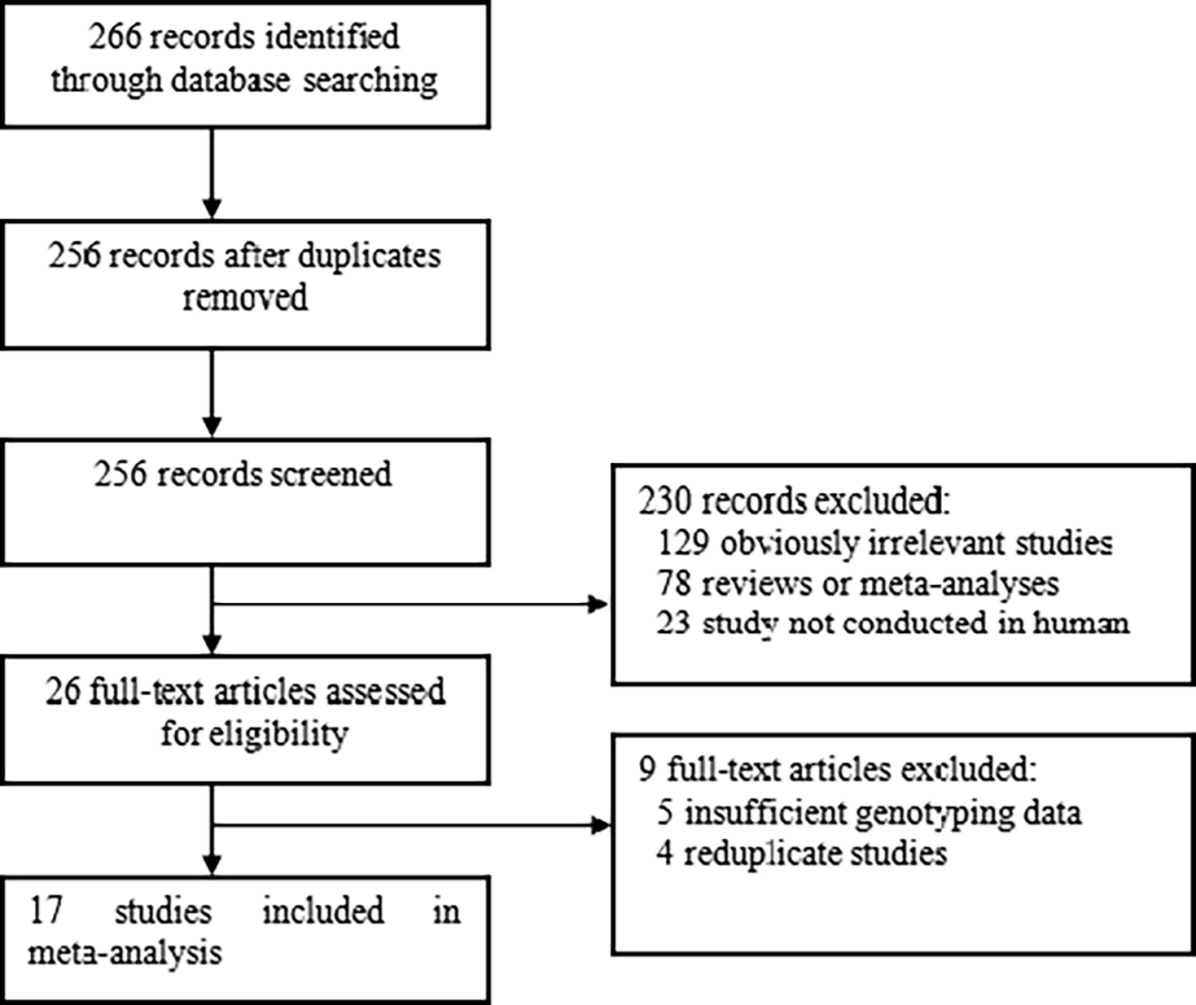
Flow diagram of the literature search.

Two hundred and sixty-six records were identified through the database search. Based on the inclusion and exclusion criteria, seventeen studies [4–20] were included, whereas 249 articles were excluded. The publication year of the involved studies ranged from 2003 to 2015. The flow chart of study selection is presented in **Fig 1**. In total, 5,756 RA cases and 4,987 controls were included in this meta-analysis. All RA patients in each study were diagnosed by the classification criteria proposed by the American College of Rheumatology for RA in 1987. Six ethnic groups, including Chinese, Japanese, British (UK), Spanish, Hungarians, and Iranians, were included in our research. Pooled analysis was not performed for the Spanish, Hungarians, and Iranians due to the presence of only one study for each of them. The characteristics of the included studies are listed in **Table 1**.

**Table 1:** Characteristics of the studies included in the meta-analysis.

References	Publication year	Ethnicity	Genotyping methods	RA number	Controls number	HWE in the controls	
						χ^2^	*P*
Cui et al. [14]	2007	China	PCR-LDR	92	116	0.03	0.852
Lu et al. [5]	2007	China	PCR-SSP	41	56	1.34	0.247
Fan et al. [6]	2008	China	PCR-SSP	70	81	7.96	0.005
Shi et al. [7]	2010	China	PCR	112	97	1.19	0.275
Feng et al. [8]	2010	China	PCR	115	106	0.28	0.595
Chen et al. [9]	2011	China	PCR	378	204	0.69	0.407
Xu et al. [10]	2011	China	PCR	130	130	0.30	0.581
Liu et al. [11]	2012	China	PCR	90	90	0.27	0.601
Li et al. [12]	2012	China	PCR-SSP	53	42	0.28	0.59
Du et al. [13]	2014	China	TaqMan	1038	1040	4.38	0.04
Suzuki et al. [4]	2003	Japan	PCR	733	735	7.42	0.01
Ikari et al. [15]	2005	Japan	PCR	1194	939	0.02	0.88
Barton et al. [16]	2004	UK	PCR	839	481	0.07	0.79
Harney et al. [17]	2005	UK	PCR	106	102	0.05	0.82
Martinez et al. [18]	2005	Spain	TaqMan	354	498	0.02	0.88
Poór et al. [19]	2007	Hungary	TaqMan	261	120	0.90	0.34
Hashemi et al. [20]	2015	Iran	PCR	150	150	0.13	0.72

### Meta-analysis

**Fig 2:**
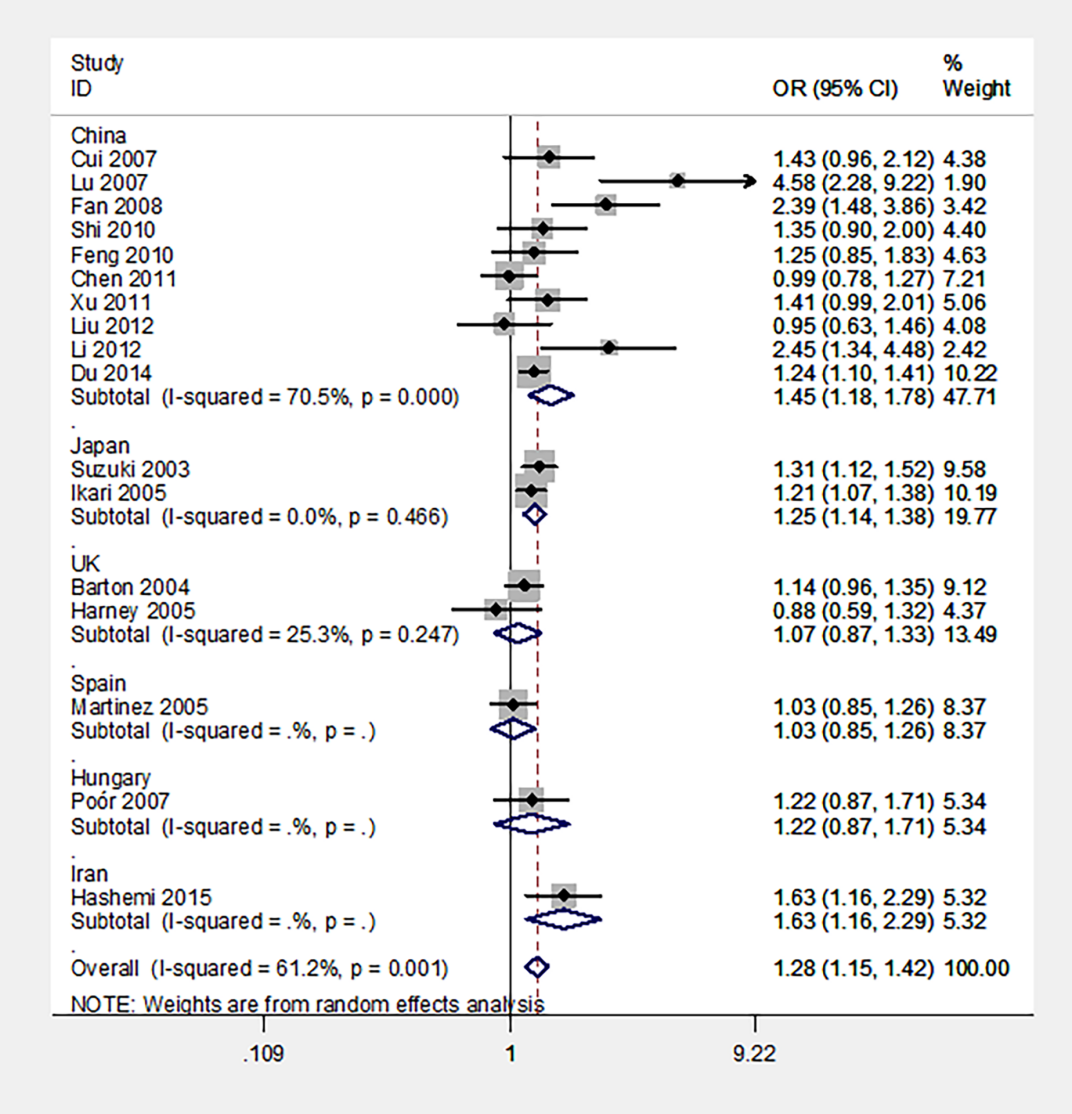
The forest plots of all selected studies on the association between PADI -104C/T polymorphism and RA susceptibility under the allele model.

The primary results of this meta-analysis concerning the association between PADI -104C/T polymorphism and RA risk are presented in Table 2. In the overall population, PADI -104C/T polymorphism was significantly associated with an increased RA risk in allele and homozygotes models, as well as with the TT versus (TC + CC), and (TT+TC) versus CC (Table 2, Fig 2).

**Table 2:** Association of the PADI -104C/T polymorphism and RA susceptibility.

Analysis model		n	OR_r_ (95% CI)	OR_f_ (95%CI)	P_h_
T vs. C	Total analysis	17	**1.28 (1.15–1.42)**	**1.23 (1.17**–**1.31)**	0.001
	China	10	**1.45 (1.18–1.78)**	**1.29 (1.18**–**1.41)**	0.000
	Japan	2	**1.25 (1.14–1.38)**	**1.25 (1.14**–**1.38)**	0.466
	UK	2	1.07 (0.87–1.33)	1.10 (0.94–1.28)	0.247
TT vs. CC	Total analysis	16	**1.50 (1.26**–**1.78)**	**1.48 (1.31**–**1.68)**	0.074
	China	9	**1.56 (1.16**–**2.10)**	**1.49 (1.24**–**1.80)**	0.094
	Japan	2	**1.68 (1.23**–**2.29)**	**1.65 (1.33**–**2.05)**	0.162
	UK	2	1.15 (0.81–1.64)	1.15 (0.81–1.64)	0.375
TT vs. CC + CT	Total analysis	16	**1.32 (1.15**–**1.51)**	**1.33 (1.18**–**1.48)**	0.232
	China	9	**1.27 (1.01**–**1.60)**	**1.28 (1.08**–**1.51)**	0.240
	Japan	2	**1.53 (1.09**–**2.15)**	**1.49 (1.22**–**1.83)**	0.099
	UK	2	1.08 (0.77–1.51)	1.08 (0.78–1.50)	0.584
TT + CT vs. CC	Total analysis	17	**1.40 (1.20**–**1.63)**	**1.31 (1.21**–**1.42)**	0.000
	China	10	**1.75 (1.30**–**2.37)**	**1.45 (1.28**–**1.65)**	0.000
	Japan	2	**1.28 (1.12**–**1.47)**	**1.28 (1.12**–**1.47)**	0.983
	UK	2	1.10 (0.81–1.51)	1.15 (0.93–1.42)	0.228

ORr: Odd ratio for the random-effects model; ORf: Odd ratio for the fixed-effects model; P_h_: *P-*value for the heterogeneity test.

#### PADI -104C/T polymorphism and RA in China and Japan

Ten studies, including 2,119 cases and 1,962 controls, identified an association between the PADI -104C/T polymorphism and RA risk in China [5–13,14], as well as two studies, including 1,927 cases and 1,674 controls, in Japan [4,15]. PADI -104C/T polymorphism was found to be significantly associated with an increased RA risk both in China and Japan in all analyzed models (Table 2, Fig 2).

#### PADI -104C/T polymorphism and RA in UK

Two studies determined the relationship between PADI -104C/T polymorphism and RA risk in UK [16–17]. The total sample size for patients with RA and controls was 945 and 583, respectively. It was revealed that PADI -104C/T polymorphism was not associated with RA in UK (Table 2, Fig 2).

### Sensitivity analysis and publication bias assessment

**Fig 3:**
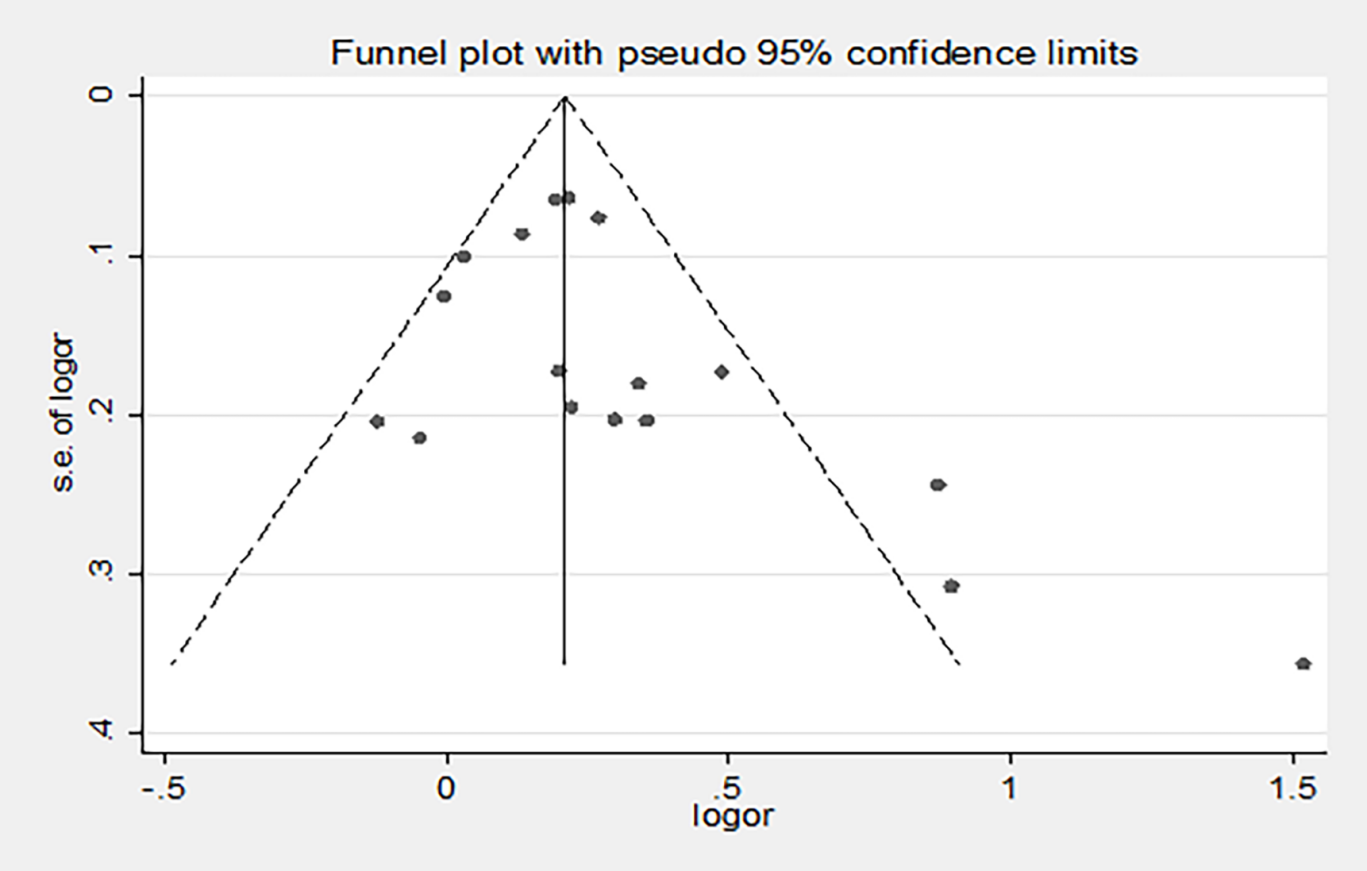
Publication bias assessment using Begg’s funnel plot.

**Fig 4:**
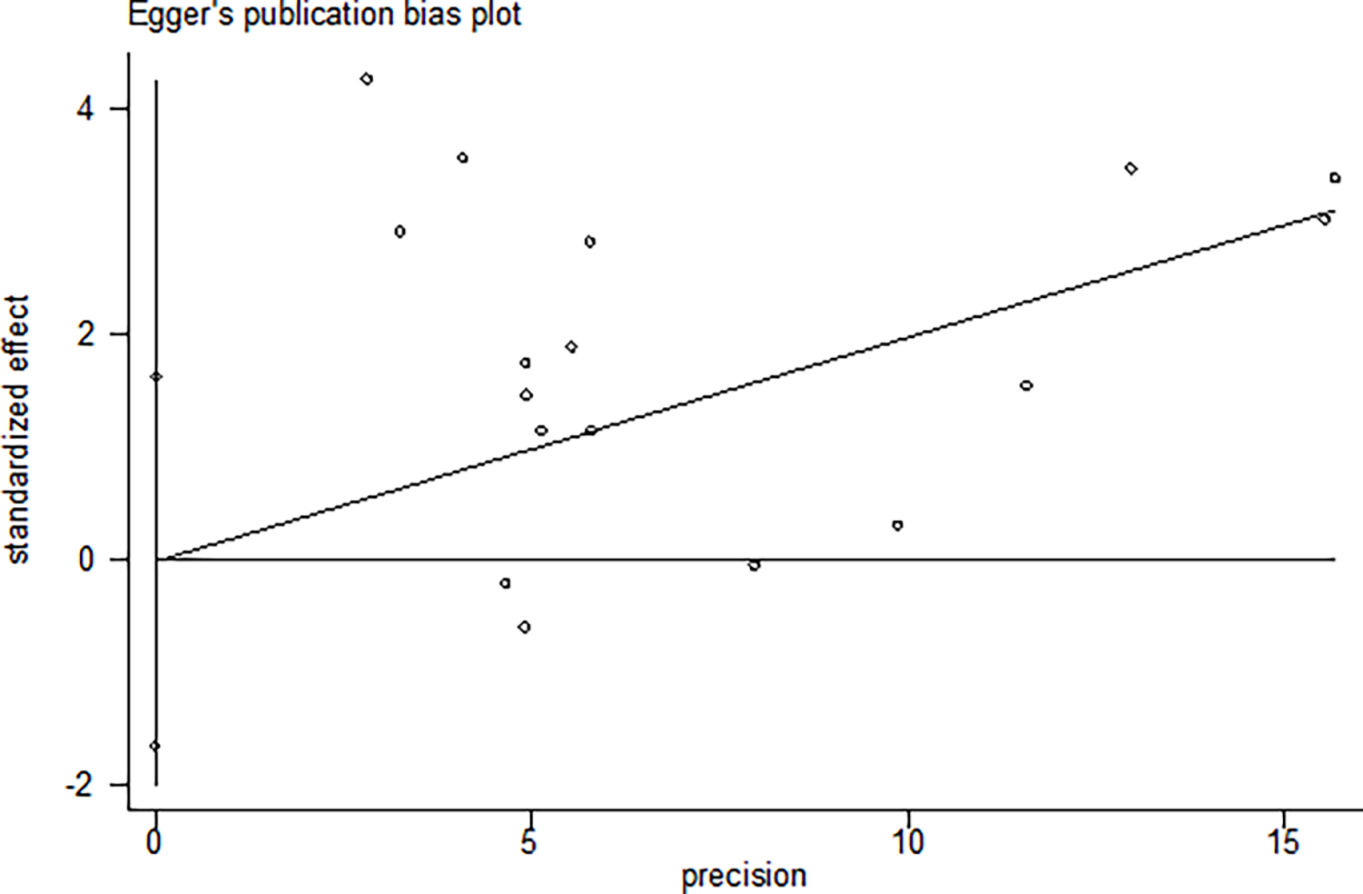
Egger’s linear regression.

To evaluate the sensitivity of this meta-analysis, we compared the consistency between the fixed-effects model and the random-effects model. The results were not materially altered, which suggests that the data in this meta-analysis were stable and reliable (**Table 2**). The Begg’s funnel plot and Egger’s test were used to evaluate the publication bias in this meta-analysis. As can be seen in Fig 3, the shape of the funnel plot did not reveal obvious asymmetry. Similarly, the Egger’s test indicated that there was no evidence of obvious publication bias in all included studies (t = -0.02, *P* = 0.984, Fig 4).

## Discussion

Although many studies have been conducted to elucidate the relationship between PADI4 -104C/T polymorphism and RA risk, a definite conclusion has not been drawn. So far, several meta-analyses have been published regarding the relationship between PADI4 -104C/T polymorphism and RA risk [21–25]. Nevertheless, their results were inconclusive and inconsistent. For example, one meta-analysis reported that there was significant association between PADI4 -104C/T polymorphism and RA risk in both an Asian and European population [21], whereas two meta-analyses found significant association only in Asian individuals [23,24]. Furthermore, the meta-analysis performed by Yang et al. [22] showed that the pooled estimates for PADI4 -104C/T polymorphism were not statistically significantly associated with RA; only one meta-analysis was conducted in a separate ethnic group [25]. Therefore, we conducted this updated meta-analysis to assess the relationship between PADI -104C/T polymorphism and RA risk in different populations. Our meta-analysis involved 17 studies with 5,756 RA cases and 4,987 controls. The results suggested that PADI -104C/T polymorphism might be a potential biomarker of RA susceptibility in the overall population. In comparison with previous meta-analyses [22–24], our research included more investigations from the Asian population, and no study was included with insufficient genotyping data.

The exact causes of ethnic discrepancy are uncertain, but studying the differences in the underlying genetic background and social factors among different populations may be important. Ethnically diverse subjects may have unique cultures and lifestyles that can contribute to different genetic characteristics and susceptibility to specific diseases. In this meta-analysis, stratified by ethnicity, we detected a significant association between PADI -104C/T polymorphism and RA risk in China and Japan. Therefore, the relationship between PADI -104C/T polymorphism and RA might be susceptible to differences in ethnicity. Because we found only one study from Spain, Hungary, and Iran, respectively [18–20], we do not discuss the association between PADI -104C/T and RA among these populations in the current meta-analysis. In comparison with previous meta-analyses [21–25], the current study involved more research in multiethnic groups. Furthermore, the effects of gene-environment interactions on RA risk were also evaluated in each separate ethnic group.

This meta-analysis is supported by an investigation of the influence of different populations on RA risk and PADI -104C/T polymorphism. The findings discussed here provide evidence for the association between PADI -104C/T polymorphism and RA risk in Chinese and Japanese populations. The sensitivity analysis and publication bias test confirmed the reliability and stability of this meta-analysis. However, only English and Chinese databases were used for the literature search in this meta-analysis, and thus other language articles/databases were not included. Therefore, further studies are needed to assess the association in other populations. In addition, the etiology of RA is complex and mediated by the activities of multiple genes. Subsequently, the effect of any single gene might have a more limited impact on RA risk than it has been anticipated so far. Finally, due to insufficient information, we could not conduct subgroup analyses stratified by other factors, such as gender, smoking status, and so on.

In conclusion, this meta-analysis indicates a significantly increased association between PADI -104C/T polymorphism and RA in Chinese and Japanese populations. Because most of the populations included in this meta-analysis were Asian, further studies are needed to elucidate if the PADI4 -104C/T gene confers RA risk in other ethnic groups.

## Supporting information

S1 File Meta analysis on genetic association studies form. (DOCX)

S2 File PRISMA 2009 checklist. (DOC)
